# Changes in Anxiety and Depression in Patients with an Acute Myocardial Infarction that Received Treatment by Percutaneous Coronary Intervention and the ABCDEFGH Bundle Including Nurse-led Continuous Follow-up

**DOI:** 10.31662/jmaj.2025-0126

**Published:** 2025-08-01

**Authors:** Tomoaki Hama, Kaho Hashimoto, Tadahiro Goto, Yuki Ozaki, Kohei Yamaguchi, Fuminobu Yoshimachi, Yuji Ikari

**Affiliations:** 1 Division of Cardiology, Department of Medicine, Tokai University Hachioji Hospital, Tokyo, Japan; 2 Division of Cardiology, Department of Medicine, Tokai University Hospital, Kanagawa, Japan; 3 TXP Medical Co., Ltd., Tokyo, Japan; 4 Department of Health Data Science, Graduate School of Data Science, Yokohama City University, Kanagawa, Japan; 5 Department of Nursing, Tokai University Hachioji Hospital, Tokyo, Japan

**Keywords:** post-intensive care syndrome, depression, anxiety, ABCDEFGH bundle, nurse-led follow-up, acute myocardial infarction

## Abstract

**Introduction:**

It remains unclear which treatments are effective for addressing mental disorders in patients with acute myocardial infarction (AMI). Therefore, we evaluated the changes over time in anxiety and depression from intensive care units (ICUs) to outpatient departments (OPDs), as well as the post-discharge prevalence of anxiety and depression in AMI patients who underwent percutaneous coronary intervention (PCI) and received treatments based on the ABCDEFGH bundle, including nurse-led continuous follow-up.

**Methods:**

This retrospective cohort study involved 29 patients who were hospitalized in the ICU for treatment of AMI. All patients received PCI and treatment based on the ABCDEFGH bundle. Anxiety and depression were assessed using the Hospital Anxiety and Depression Scale (HADS) at three phases: ICU, general ward (GW), and OPD. First, we compared changes in anxiety and depression scores over time using HADS across the three phases. Second, we assessed the prevalence rate of anxiety and depression after hospital discharge based on HADS scores.

**Results:**

Of the 29 patients, the mean age was 67 ± 14 years, and 66% were male. For the anxiety subscale (HADS-A), scores improved from ICUs to OPDs (6.4-3.9 points, p = 0.001). For the depression subscale (HADS-D), there were no significant changes between ICU and GW, or between ICU and OPD (5.4-6.7, 5.4-5.2 points, respectively; all not significant). At the OPD stage, 14% of patients were in an anxiety state, and 34% were in a depression state.

**Conclusions:**

In AMI patients who received PCI and treatments based on the ABCDEFGH bundle, the depression state remained unchanged from ICUs to OPDs, while the anxiety state improved after hospital discharge. The prevalence of anxiety was 14%, and the prevalence of depression was 34% after hospital discharge.

## Introduction

Many severe illnesses have afflicted patients in intensive care units (ICUs) for many years. Acute myocardial infarctions (AMIs) remain one of the high-mortality diseases among critical illnesses. However, in recent years, the in-hospital mortality rate for AMI has decreased dramatically due to shorter arrival times at hospitals, advances in reperfusion therapy (such as coronary artery bypass grafting and percutaneous coronary intervention [PCI]), and improvements in ICU treatment. As a result, the number of survivors who maintain a high level of activities of daily livings at the time of discharge has increased ^(1)^. Therefore, many AMI survivors suffer from mental health disorders, particularly anxiety and depression ^(2), (3), (4)^.

Even worse, depression in patients after an AMI could be a significant risk factor for mortality ^(5), (6)^. Although we should actively intervene in these mental disorders, it is unknown which treatments are effective. Therefore, AMI patients who were hospitalized in the ICU were treated by optimal therapy in addition to the ABCDEFGH bundle in this hospital. The bundle is recommended by professional critical care organizations to help reduce post-intensive care syndrome (PICS), including anxiety and depression ^(7), (8)^. It consists of **A**irway management, **B**reathing trials, **C**oordination of care and **C**ommunication, **D**elirium assessment, Early mobility, **F**amily involvement, **F**ollow-up referrals, **F**unctional reconciliation, **G**ood handoff communication, and **H**andout materials ^(9)^.

To the best of our knowledge, no studies have reported the changes over time in anxiety and depression from ICUs to outpatient departments (OPDs), or the post-discharge prevalence of anxiety and depression in AMI patients treated by PCI and the ABCDEFGH bundle. We aimed to evaluate how anxiety and depression changed from ICUs to OPDs and to assess the prevalence of anxiety and depressive disorders after hospital discharge in patients hospitalized in ICUs for AMI treatment with PCI and the ABCDEFGH bundle.

## Materials and Methods

### Study design and setting

This was a retrospective cohort study using data from patients who were admitted to Tokai University Hachioji Hospital and stayed in the ICU for evaluation and treatment of AMIs from July 2019 to January 2021. This study was conducted in accordance with the Declaration of Helsinki and approved by the Research Ethics Committee of Tokai University (23R-242). Written consent has been obtained from all patients (or legal guardians) to publish personal information.

### Participants

We identified adult patients (aged ≥20 years) who were treated for an AMI in ICUs with the ABCDEFGH bundle. AMIs were defined as a typical rise and gradual fall (troponin) or a more rapid rise and fall (creatine kinase MB isoenzyme) in biochemical markers of myocardial necrosis, along with ischemic symptoms or electrocardiogram changes indicative of ischemia (ST elevation or depression) ^(10)^. In addition, all patients received PCI to treat the AMI and treatment based on the ABCDEFGH bundle. Their anxiety and depression state were measured in three phases: ICUs, general wards (GWs), and OPDs.

### Outcomes

We measured the anxiety and depression states using the Hospital Anxiety and Depression Scale (HADS) ^(11)^. The HADS has been used in numerous research studies on mental disorders for many years. Especially in the cardiovascular field, previous studies have used the HADS to provide evidence of the association between cardiovascular diseases and mental disorders ^(12), (13)^. The scale consists of 14 items: seven items are subscales to assess anxiety (HADS-A), and the other seven items are subscales to assess depression (HADS-D). Scores of 0-7 indicate no anxiety or depressive disorder; scores of 8-10 indicate moderate anxiety or depressive disorder; scores of 11 or higher indicate high anxiety or depressive disorder. One research study reported that the HADS-A specificity is 0.78 and sensitivity is 0.9, while the HADS-D specificity is 0.79 and sensitivity is 0.83 ^(11)^. In this study, the anxiety and depression states were defined as subscale scores of 8 or higher. The HADS assessments were conducted by expert nurses. Specifically, HADS in the ICU was evaluated by ICU nurses, HADS in the GW was evaluated by GW nurses, and HADS in the OPD was evaluated by OPD nurses.

### Measurements

All data were abstracted from medical records in the hospital. The patient medical records were retrospectively reviewed, and the investigational items were collected, including age, sex, job before admission, coronary risk factors (smoking, hypertension, diabetes mellitus, and dyslipidemia), medications during the ICU stay (intravenous sedative drugs, oral tranquilizers and intravenous pain killers), mechanical support (mechanical ventilations and insertion of Intra-Aortic Balloon Pumping), blood examinations (triglyceride, low density lipoprotein-cholesterol, high density lipoprotein-cholesterol, glycated hemoglobin A1c, brain natriuretic peptide, hemoglobin, albumin, estimated glomerular filtration rate, peak creatine kinase [CK], peak CK MB isoenzyme [CK-MB], troponin T, and C-reactive protein, echocardiography (left atrial diameter, left ventricular diastolic diameter, and left ventricular ejection fraction), Sequential Organ Failure Assessment (SOFA) score at ICU admission and discharge, Intensive Care Delirium Screening Checklist (ICDSC) during the ICU stay, Mini Mental State Examination (MMSE) in the three phases, use of ICU diaries, psychiatrist intervention, online visits with family members, duration of ICU stay, time from ICU admission to getting out of bed, duration of hospitalization, and time from hospital discharge to the nurse interview using the HADS in the OPD. We eliminated patients who did not undergo PCI or who were not assessed using the HADS at all three time points, including those who died or were transferred during the observation period.

### The ABCDEFGH bundle, including nurse-led continuous follow-up

The ABCDEFGH bundle is part of the new guidelines on pain, agitation, and delirium published by the Society of Critical Care Medicine ^(9)^. The bundle was applied to AMI patients who stayed in the ICU of this hospital, and the details are as follows. **A**, **B**, and **C** represent *airway management, spontaneous breathing trials, and coordination of care and communication among disciplines*. Professional ICU medical staff routinely attempted to stop sedation and remove intubation tubes from patients who were sedated or intubated. **D** represents *delirium assessment and prevention*. In this hospital, delirium was evaluated using the ICDSC ^(14)^. When patients were diagnosed with delirium and medical staff assessed that professional treatment was needed, they consulted psychiatrists for patient care. In addition, all patients with AMI were monitored by a clinical psychotherapist as part of the AMI treatment. **E** stands for *early mobility*. In this hospital, a professional team for early ambulation routinely assessed patients’ ability to stand and walk as early as possible. **F**, **G**, and **H** stand for *family involvement, follow-up referrals, functional reconciliation, good handoff communication, and providing the patient and family written information about the elements of PICS*. To implement the FGH components of the bundle, regular medical conferences were held with expert nurses from three departments: ICU, GW, and OPD. The team members included certified nurses in Intensive Care, Chronic Heart Failure Nursing, and Dementia Nursing. In those conferences, the nurses carefully exchanged patient information, including the patient’s mental health status, using the HADS. If the nurses determined that additional interventions for the patients’ mental disorders were necessary, they arranged for psychiatrists or clinical psychologists to intervene and/or recommended the use of an ICU diary for the patients and their families ^(8), (15), (16)^.

### Statistical analysis

Data are presented as the mean ± standard deviation or number (%) as appropriate. A paired t-test was used to compare the HADS subscales (HADS-A or HADS-D) between ICU and GW, and ICU and OPD. All tests were evaluated at a significance level of p < 0.05. Statistical analyses were performed using SPSS for Windows Version 16.0 software (SPSS Inc., Chicago, IL, USA).

## Results

### Clinical characteristics of the participants

The demographics and clinical information of the 29 eligible participants are provided in Table 1. The mean age was 67 years, and 66% were male. Fourteen patients (14/29, 48%) had a job before admission. About 50% of the patients had a history of smoking. One patient took oral tranquilizers (Zolpidem) during hospitalization. No patients received intravenous sedative drugs or painkillers. There were no intubated patients. The patients’ lipid profiles and diabetes management were well controlled. The mean left ventricular systolic function in the patients was preserved. The mean SOFA scores were 1.4 points at ICU admission and 1.3 points at ICU discharge. In the ICDSC, 3 patients had 2 points, 3 had one point, and the others had 0 points. The patients maintained nearly normal cognitive function as estimated by the MMSE during the observation periods. No patients or families used ICU diaries in this study. One patient received professional treatment from a psychiatrist. Due to the coronavirus disease 2019 (COVID-19) pandemic, 38% of patients had online visits with their families. The average ICU stay was 4.2 days. It took the study patients a mean of 2.8 days to get out of bed.

**Table 1.** The Baseline Characteristics of 29 Patients with an Acute Myocardial Infarction That Had Been Hospitalized in ICUs.

Variables	Overall
	(n = 29)
**Patient demographics**
Age (years)	67 ± 14
Male sex	19 (66%)
Job before admission	14 (48%)
Coronary risk factors
Smoking (current and ever)	16 (55%)
Hypertension	12 (41%)
Diabetes mellitus	7 (24%)
Dyslipidemia	7 (24%)
Medications in ICUs
Intravenous sedative drugs	0 (0%)
Oral tranquilizers	1 (3%)
Intravenous pain killers	0 (0%)
Mechanical supports
Mechanical ventilations	0 (0%)
Insertion of IABP	4 (14%)
**Blood examinations**
Triglyceride (casual) (mg/dL)	126 ± 72
LDL-cholesterol (mg/dL)	123 ± 38
HDL-cholesterol (mg/dL)	52 ± 13
HbA1c (%)	5.9 ± 0.4
BNP (pg/mL)	424 ± 774
Hemoglobin (g/dL)	13.6 ± 2.0
Albumin (g/dL)	3.8 ± 0.5
eGFR (mL/min/1.73m^2^)	71.0 ± 23.3
Peak CK (IU/L)	2123 ± 1951
Peak CK-MB (IU/L)	198 ± 164
Troponin T (ng/mL)	5.180 ± 8.126
CRP (mg/L)	1.905 ± 2.647
**Echocardiographic findings**
LAD (mm)	37 ± 5
LVDd (mm)	48 ± 4
LVEF (%)	52 ± 7
**SOFA score**
Entering the ICUs (points)	1.4 ± 2.0
Leaving the ICUs (points)	1.3 ± 1.2
**ICDSC**
Staying in the ICU (points)	0.3 ± 0.6
**MMSE**
Staying in the ICU (points)	27.0 ± 2.1
Staying in the GW (points)	29.1 ± 1.5
Staying in the OPD (points)	28.7 ± 1.6
**The use of ICU diaries**	0 (0%)
**The intervention of a psychiatrist**	1 (3%)
**The online visits with family members**	11 (38%)
**The period of time**
The duration of the ICU stay (days)	4.2 ± 4.9
The duration from admission to the ICU to leaving the bed (days)	2.8 ± 4.0
The duration of hospitalization (days)	13.7 ± 13.7
The duration from hospital discharge to the nurse’ interview with the HADS in OPDs (days)	76.3 ± 75.4

Data are presented as the mean ± SD, or n (%). Abbreviations: CK: creatine kinase; CK-MB: creatine kinase MB isoenzyme; CRP: C-reactive protein; eGFR: estimated glomerular filtration rate; GW: general ward; HADS: Hospital Anxiety and Depression Scale; IABP: intra-aortic balloon pumping; ICDSC: Intensive Care Delirium Screening Checklist; ICU: intensive care unit; LAD: left atrial diameter; LVDd: left ventricular end-diastolic diameter; LVEF: left ventricular ejection fraction; MMSE: Mini Mental State Examination; OPD: outpatient department; SOFA: Sequential Organ Failure Assessment.

### The time changes in the anxiety and depression state and prevalence of moving from ICUs to OPDs

**Figure 1.**
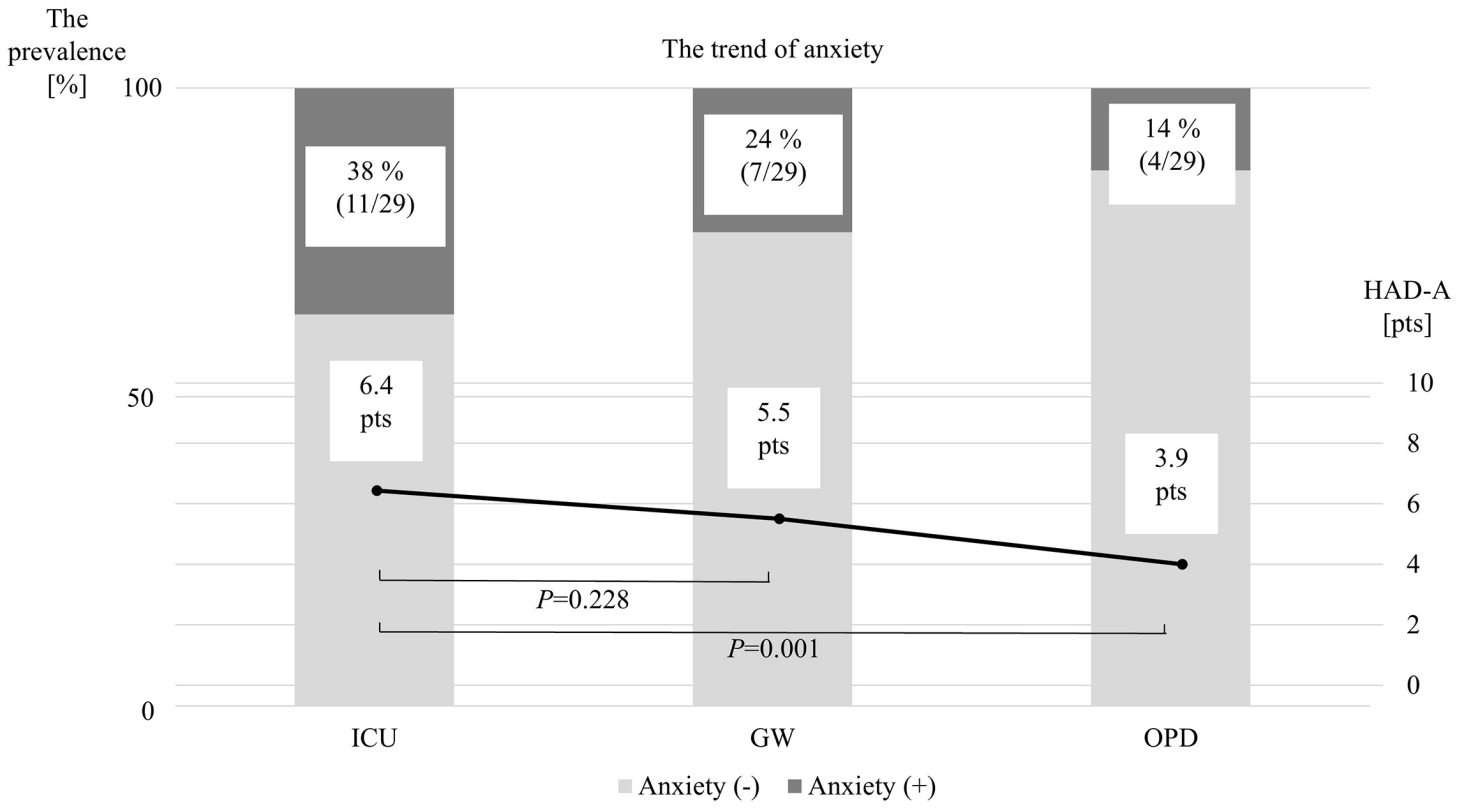
The time changes in the prevalence rate of anxiety and the HADS-A among the three phases. HADS-A: Hospital Anxiety and Depression Scale-Anxiety.

**Figure 2.**
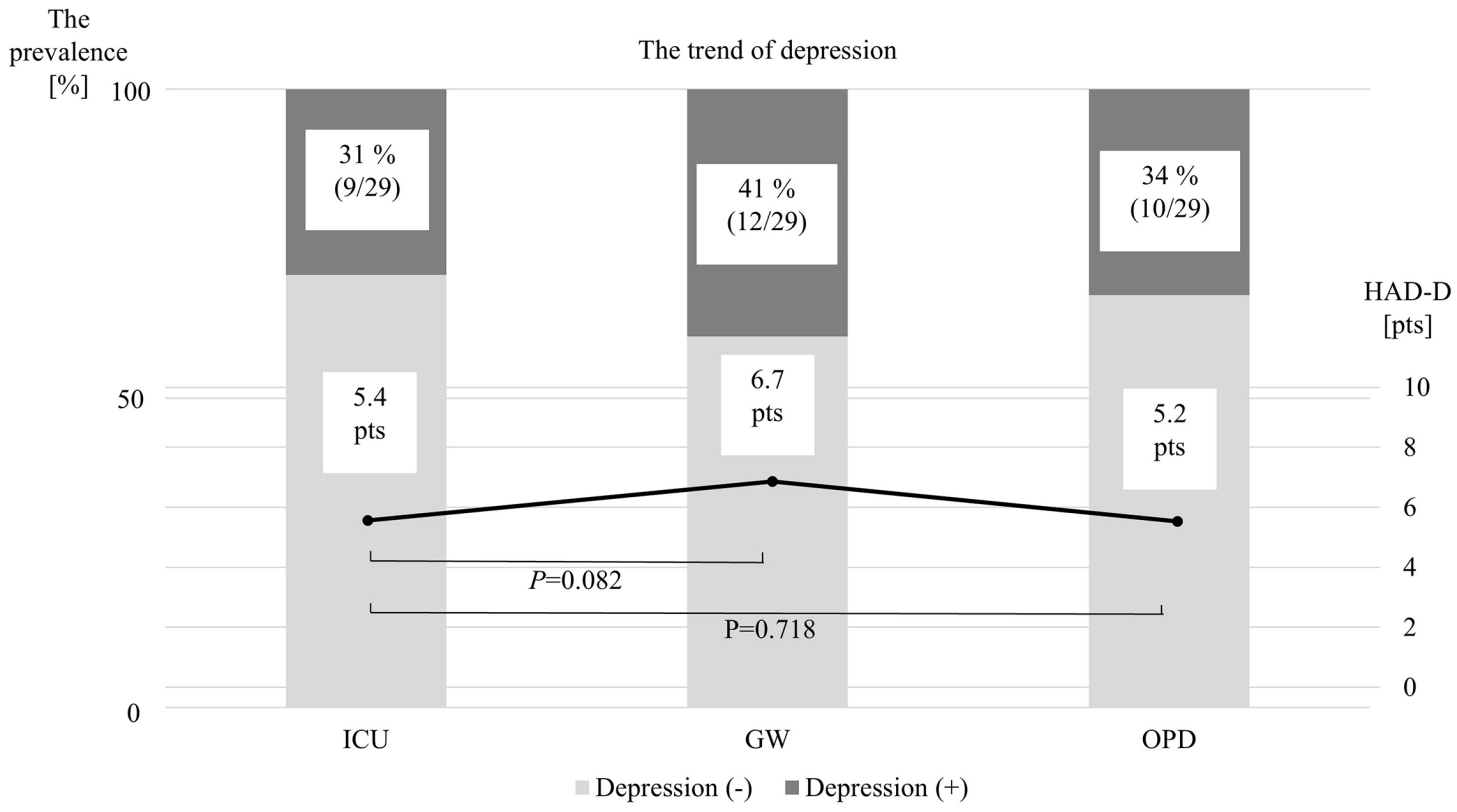
The time changes in the prevalence rate of depression and the HADS-D among the three phases. GW: general ward; HADS-D: Hospital Anxiety and Depression Scale-Depression; ICU: intensive care unit; OPD: outpatient department.

Table 2 and Figure 1 show the changes in the anxiety subscales (HADS-A) and prevalence from ICUs to OPDs. During their stay in the ICUs, the mean HADS-A score was 6.4 ± 3.6 points, and 11 patients were in an anxiety state (11/29, 38%). When they moved to the GWs, the mean HADS-A score was 5.5 ± 3.0 points, and 7 patients were in an anxiety state (7/29, 24%). The subscale did not significantly change from ICUs to GWs (p = 0.228). When they moved from ICUs to OPDs, the HADS-A score significantly decreased to 3.9 ± 3.0 points from the ICU period (p = 0.001), and only 4 patients were in an anxiety state (4/29, 14%). On the other hand, the changes in the depression subscales (HADS-D) and prevalence from ICU to OPD are shown in Table 2 and Figure 2. When they were in the ICUs, the HADS-D score was 5.4 ± 3.6 points, and 9 patients were in a depressive state (9/29, 31%). When they moved to the GWs, the HADS-D was 6.7 ± 4.3 points, and 12 patients were in a depression state (12/29, 41%). When they were discharged to the OPD, the HSDS-D was 5.2 ± 3.6 points, and 10 patients were in a depressive state (10/29, 34%). Statistically, there were no differences in HADS-D between the ICU and GW, or between the ICU and OPD.

**Table 2.** The Time Changes in the HADS-A and HADS-D, and Prevalence Rate of Anxiety and Depression among the Three Phases.

Outcomes (n = 29)	Phase1. ICU	Phase2. GW	Phase3. OPD	p Value	
				Phase 1 vs Phase 2	Phase 1 vs Phase 3
HADS-A (pts)	6.4 ± 3.6	5.5 ± 3.0	3.9 ± 3.0	0.228	0.001
Prevalence rate of anxiety (%)	38% (11/29)	24% (7/29)	14% (4/29)		
HADS-D (pts)	5.4±3.6	6.7±4.3	5.2±3.6	0.082	0.718
Prevalence rate of depression (%)	31% (9/29)	41% (12/29)	34% (10/29)		

Data are presented as the mean ± SD, or % (n). GW: general ward; HADS: Hospital Anxiety and Depression Scale; HADS-A: Hospital Anxiety and Depression Scale-Anxiety; HADS-D: Hospital Anxiety and Depression Scale-Depression; ICU: intensive care unit; OPD: outpatient department; pts: patients.

## Discussion

In this study of 29 AMI patients who received PCI and treatment based on the ABCDEFGH bundle, we found that the anxiety state measured by the HADS-A improved, while the depression state measured by the HADS-D remained after hospital discharge. Additionally, the prevalence after hospital discharge was 14% for the anxiety state and 34% for the depression state. To the best of our knowledge, this is the first study to report the time changes and prevalence of anxiety and depression states from the ICU to the OPD in AMI patients who were treated with PCI and the ABCDEFGH bundle.

Several studies have demonstrated the prevalence of anxiety after hospital discharge in patients with AMIs. The prevalence of anxiety disorder varied widely in previous studies (4.5%-52%) ^(3), (4), (13)^. In light of past studies, the results of this study could indicate that the prevalence of anxiety was relatively lower than in those studies (14%). One reason for the low prevalence in this study might have been the limited presence of risk factors causing anxiety. In other words, although previous studies showed that predictors of anxiety after recovery from critical illnesses included being female, younger age, and duration of mechanical ventilation in critical care, many of the patients in this study were elderly and male, and no patients were intubated ^(17), (18)^. In addition, the interventions based on the ABCDEFGH bundle might have affected their anxiety state. Regarding the time change in anxiety, one previous research study showed the time change in the prevalence of an anxiety state over the course of one year ^(3)^. The research showed that an improvement in anxiety was observed before hospital discharge, but the prevalence gradually increased after hospital discharge. Conversely, in this study, the anxiety subscales improved after hospital discharge. The previous research considered that the improvement before hospital discharge was caused by the disappearance of distressing symptoms of ischemic heart disease, and the deterioration after hospital discharge was due to recognition of severe cardiovascular disease and impaired functional capacity. We extrapolated that one reason the anxiety subscales gradually decreased from the ICU to the OPD in this study was that the incidence of anxiety disorder might have been suppressed by the continuous follow-up of expert nurses from the ICU to the OPD.

The prevalence of depression was shown to be about 20 to 40% in several previous studies ^(2), (3), (12)^. One systematic review indicated that major depression in survivors of an AMI was identified in 19.8% of patients ^(19)^. The prevalence in the present study was similar to those results (34%). Previous studies concluded that predictors of depression in patients with critical illnesses were female, younger age, sedation, psychiatric symptoms at discharge, and impairment of physical function ^(17), (18)^. Although there were a few risk factors for the incidence of depression in the patients in this study, the prevalence did not improve after hospital discharge. The mechanisms of the onset of depressive symptoms in AMI patients are unknown. However, the following might explain the results of this study. It is well known that AMI patients are usually exposed to a hypoxic environment during the acute phase ^(20)^. Further, a previous study indicated that biological factors, including cerebral hypoxia, could contribute to a depressive state in patients undergoing treatment in the ICU ^(18), (21)^. For this reason, the depression subscales and prevalence in these study patients might not have improved from the ICU to the OPD.

Although the ABCDEFGH bundle is well known as a group of interventions that addresses the risks of sedation, delirium, and immobility, it is unknown what treatment should be provided to patients with critical illnesses who stay in the ICU to prevent and treat anxiety and depressive disorders. One study showed that early rehabilitation did not improve patients’ mental status-related outcomes ^(22)^. However, the part we would like to emphasize is that the bundle includes the ABCDE bundle and additional components of the ‘FGH’ bundle (*family involvement, follow-up referrals, functional reconciliation, good handoff communication, and providing the patient and family with written information about the elements of PICS*). In other words, a transition out of the ICU is important for intervening in mental health disorders ^(9)^. Several studies have indicated that the FGH bundle, including improving communication, providing family support, family presence in the ICU, and professional psychological intervention, could mitigate mental health disorders ^(23), (24), (25)^. Furthermore, Nagaya et al. ^(26)^ emphasized the continuation of the ABCDE bundle, i.e., the utilization of the FGH bundle, because it is vital for addressing multiple aspects of immobility and delirium during the ICU stay. Although the PRaCTICaL study did not show that the nurse-led intensive care follow-up program was effective in improving the long-term outcomes from critical illness, post-ICU follow-up might be essential for communication with patients, their relatives, ICU team staff, and psychology specialists ^(27)^. In the present study, one reason the anxiety subscales improved after hospital discharge might be that expert nurses in three departments continuously exchanged patient information and followed up on mental status from the ICU to the OPD across department boundaries.

The most important finding of this study was the possibility of a beneficial effect of the ABCDEFGH bundle, including nurse-led follow-up, in addition to traditional treatment in AMI patients. It remains unknown whether the ABCDEFGH bundle can be effective for mental disorders in AMI patients. Moreover, to the best of our knowledge, this is the first study to report on PCI and treatment based on the ABCDEFGH bundle, including nurse-led continuous follow-up, in AMI patients. Even now, many patients with AMIs are still suffering from anxiety and depressive disorders. Our findings might facilitate more AMI patients receiving treatment based on the ABCDEFGH bundle, as the treatment could mitigate a worsening of their anxiety and depression state.

### Potential limitations

This study had several potential limitations. First, the sample size, especially the number of patients in a severe critical state (e.g., use of mechanical supports, intravenous sedative drugs, and a high SOFA score), was relatively limited, although this is one of the largest studies to have investigated time changes and prevalence in anxiety and depression among AMI patients treated with PCI and the ABCDEFGH bundle. Second, there might have been unmeasured confounding factors affecting the time changes and prevalence due to the retrospective design. Third, we did not include a control group of patients who received only traditional treatment without the ABCDEFGH bundle. Fourth, the timing of the HADS measurement varied considerably among participants. Fifth, we did not collect any long-term follow-up data. Therefore, the time change in the HADS and the prevalence in the long term remain unclarified. Sixth, HADS was evaluated by nurses belonging to each department, so there might have been differences in quality between nurses working within the same department and between departments. In addition, some patients might not have received treatment involving “family involvement” as part of the bundle because visits with family members were restricted to online-only during the COVID-19 pandemic. Lastly, the single-center design limited the generalizability of the current findings. Thus, further studies are needed to validate the current findings.

### Conclusions

In AMI patients who underwent PCI and additional treatment based on the ABCDEFGH bundle, including nurse-led continuous follow-up, the anxiety state improved after hospital discharge, and the prevalence of anxiety was 14%. On the other hand, the depression state remained unchanged from the ICU to the OPD, and the prevalence of a depression state was 34% after hospital discharge. Our findings should facilitate further prospective randomized controlled trials to verify the effect of the ABCDEFGH bundle on mental disorders in AMI patients.

## Article Information

### Conflicts of Interest

None

### Acknowledgement

The authors express their special gratitude to Mr. John Martin for the English language review.

### Author Contributions

Tomoaki Hama had full access to all the data in the study and takes responsibility for the integrity of the data and the accuracy of the data analysis. Concept and design: All authors. Acquisition, analysis, or interpretation of data: Tomoaki Hama, Kaho Hashimoto, Yuki Ozaki, and Kohei Yamaguchi. Drafting of the manuscript: Tomoaki Hama, Kaho Hashimoto, and Tadahiro Goto. Critical revision of the manuscript for important intellectual content: All authors. Statistical analysis: Tomoaki Hama, and Kaho Hashimoto. Administrative, technical, or material support: Tomoaki Hama, Yuki Ozaki, and Kohei Yamaguchi. Supervision: Tadahiro Goto, Fuminobu Yoshimachi, and Yuji Ikari.

### Approval by Institutional Review Board (IRB)

IRB approval code issued: 23R-242; The name of the institution that granted approval: The research ethics committee of Tokai University.

### Availability of Data and Materials

The data underlying this article will be shared on reasonable request to the corresponding author.

### Meeting Presentation

The paper was presented at the Clinical Research Award session of the 272nd Meeting in the Kanto-Koshinetsu Region of the Japanese Circulation Society; June 1, 2024; Tokyo, Japan.
